# The perplexities of a pandemic: A black Canadian scholar’s perspective

**DOI:** 10.1177/1473325020973294

**Published:** 2021-03

**Authors:** Akin Taiwo

**Affiliations:** School of Social Work, King’s University College at Western University, London, Ontario, Canada

**Keywords:** Reflective practice, pedagogy, resilience

## Abstract

Covid-19 presented an existential and ontological threat to a Black professor of Social Work at a liberal arts college in Canada. This paper is a reflective account of the tremendous shift in gear regarding his pedagogy and vulnerabilities and their implication for social work education and the resilience of his students.

I did not want to admit to a pandemic. I could not say that was denial. Though I have taught several social work classes touching on globalization, I did not think that the Corona Virus could touch us so quickly in Canada. Maybe that was wishful thinking. In some of my classes, we have talked about the swift movement of goods and services across national borders as well as the internationalization of local conflicts, wars, and diseases (Dominelli, 2010). We have even talked about the Bubonic plague that decimated Europe and gave rise to the Statute of Labourers in 1351 England as we tried to understand the history of social work as an international profession (Hick and Stokes, 2017; Poos, 1983). I did not realize that we were standing on the verge of another historic event, a raging pandemic in the neoliberal era. Maybe I was in denial, after all, with a dose of magical thinking.

Tagged as Covid-19, the pandemic unambiguously came to us in March of 2020. Canada shut down within a week as my college shut down within three days of a national emergency announcement. It caused such a huge disruption to the academic session as we had three working days to transition every class online. It was not a time to think of pedagogy for those who were not already invested in online education. Rather, it was a time to quickly react or respond to the immediate needs of students to complete the semester for which they had already paid. The pandemic was both disruptive and distracting, but it also gave birth to new discoveries, not least the resilience of students in the face of a public and international health crisis.

## Confronting myriad fears

How do you fight an unseen and unknown enemy? Our global village has a virus that was largely unpredictable, yet virulent and contagious, with high morbidity and mortality rates. It was no longer abstract or distant but concrete, close, and ominous. Many lives were threatened by it, including my students’ and mine, making the pandemic personal, thereby propelling fear in us. Moreover, the science of the virus has not been exact. The infection could be transmitted by a carrier who is symptomatic or asymptomatic or it could be contained in a carrier and not caught by family members. It could also be airborne or contracted through the common doorknob. The reality was overwhelming for all of us as we read the constant barrage of statistics of infections, deaths, and dying in popular media. Yet, we could not look away from the pandemic that deepened the fear of others, and exacerbated inequality, inequity, unemployment, and poverty around the world.

Economically speaking, the pandemic disrupted the global supply chain and made me realize how dependent Canada has been on goods and services from China and the United States of America. Hockey players initially cried foul about the shortage of hockey sticks, not knowing that the country would soon lament the shortage of respiratory masks (Osman, 2020; Whyno, 2020). There was an immediate decline in economic activities as many companies shut down or moved their operations online. This caused multiple job losses and financial stress for my immediate constituents, students who were working to sustain themselves and their families. This labour market situation practically opened my eyes to the difficulties of modern students who could no longer just be students but were also workers, parents, or care providers.

Furthermore, the pandemic caused a disruption to the academic routine I thought I was mastering. It was when classes moved online that I realized that several students did not have personal computers or reliable internet connectivity but had been using library resources. This affected the motivation of some as they had to scramble to complete their courses in ways not previously anticipated. Yet, some students thrived online, achieving better grades and finishing their semester stronger as they worked at their own pace. I had to deconstruct and dispel the discourse of fear in my online meetings with students.

To prepare for my first (pandemic) class, I only had to do voice-overs on my PowerPoint presentations so that students could listen asynchronously. As a Black professor with a distinct African accent, this exercise created a certain vulnerability in me. Talking in class was never problematic for me as I was good at reading students’ body language and social cues, but ‘teaching blind’ without student input became suddenly scary. Would students understand my accent without being able to read my lips or follow my mannerisms? Would they care enough to learn the course content? I had to confront the fear of being misunderstood by recording the lectures as best as I could. I encouraged students to join me on zoom for office hours where I would answer questions and clarify problematic concepts. However, not a single student expressed any difficulty with my accent or enunciation. I think they extended grace at a moment of teacher vulnerability. In turn, I expressed thanks to the universe for this gracious gesture.

Nevertheless, for a profession that thrives on interaction, isolation is not pedagogically compatible with social work education. Students felt trapped at home and some expressed that they missed human connections. Some became restless as they felt secluded, and many became exhausted by suddenly having free time on their hands. As expressed to me in my zoom classes, the pandemic started taking a toll on students’ emotional and mental health. For instance, the feeling of anxiety was commonplace, causing a distraction to the mastery of course materials for many students. Some students reported feeling worn-out emotionally, not sleeping well, not able to stop worrying, having to be on high alert, and feeling hopeless and depressed.

Furthermore, the feeling of uncertainty also caused many students to binge on the news and social media and on certain television programs. I advised students to ensure that their new distractions did not become a habit or an addiction. To this end, my zoom office hours literally became group therapy sessions though students were not supposed to be my clients. I was just being human with them, acknowledging uncertainty, expressing empathy, sharing vulnerabilities, and being as honest as possible regarding my own feelings. At the official end of my classes, students requested that I should continue holding weekly zoom meetings with them where they could check-in until the end of their examination. They expressed that I had a calming effect on them. I embraced this idea and ‘stayed’ with them until the end of the semester.

## Distractions and discoveries

It is imperative to note that there was an immediate backlash of racism and xenophobia against Asian Canadians, especially the Chinese, at the initial spread of the virus. This was a great distraction from the public health crisis. I challenged my students to confront such distraction and discrimination at the risk of becoming unpopular with their friends or family members who might hold such prejudicial attitudes. I pointed out that Chinese Canadians were, as all other people, victims of the pandemic notwithstanding the vituperations of some uninformed folks within and beyond Canada. For social work students who already knew that scapegoating certain individuals within the family during therapy or groups of people within the society never resolved a crisis, this became a veritable teachable moment.

On a more personal note, as a Black man, I have often observed some White people cross the road to the other side when I walked towards them on my evening strolls. During this period of social distancing, I have not felt awkward anymore seeing them exhibit this same behavior. Trying to relate this observation to course content, one of my students interpreted my experience as “racism as a protective factor” in the course of the pandemic. I am still reflecting on that notion.

On a different note, regarding discoveries, I had never heard about zoom until the pandemic, and I learned about this platform to deliver the rest of my lectures online. Though I am not a tech-savvy professor, I discovered that I could learn quickly alongside most of my students. Zoom also became the platform to dispense hope and encouragement amid the uncertainty faced by many. I discovered that my students needed a bit of handholding until they became steady on their feet, a need I was able to fill.

At the macro level, Canada established the emergency response benefit programs to reduce the financial anxiety of her citizens. This consisted of cash transfers to the recently unemployed, including students and small businesses. I discovered that the discourse of neoliberalism and the free market system was muted, indicating that governments have no qualms about tending towards ‘socialism’ in moments of great crisis, though big businesses still had a way of not being left out of the largesse of state, especially as demonstrated by the distribution of the trillion-dollar financial aid package in the USA. I used this observation in my social policy class to illustrate the need for the transformation of the whole socio-economic structure into that which is more equitable.

Lastly, I was amazed about the resilience of my students during the pandemic. They were able to resourcefully adapt despite their stressful experiences. Though there was an initial panic, they were all able to negotiate and navigate their different environments to successfully complete their semester. They showed flexibility as we recalibrated and renegotiated last assignments and group presentations. We all demonstrated the inter-relatedness of our lives and our common humanity. Though normalcy may still be an eternity away, especially with the added burden of racial tumult that gripped the attention of the world, I still hold out the hope that tomorrow shall be a better, healthier day for us all, where our governments are better prepared to handle any pandemic and our educational sector reimagined to better accommodate the emerging needs of students.

## References

[bibr1-1473325020973294] Dominelli L (2010) Globalization, contemporary challenges, and social work practice. International Social Work 53(5): 599–612.

[bibr2-1473325020973294] Hick S Stokes J (2017) Social Work in Canada: An Introduction. 4th ed. Toronto: Thompson Educational Publishing.

[bibr3-1473325020973294] Osman L (2020) Coronavirus: Canadian doctors unsure when they’ll see newly arrived masks. Available at: https://globalnews.ca/news/6835258/coronavirus-canada-doctors-mask-shortages/ (accessed 29 October 2020).

[bibr4-1473325020973294] Poos LR (1983) The social context of statute of labourers enforcement. Law and History Review 1(1): 27–52.

[bibr5-1473325020973294] Whyno S (2020) Amid virus outbreak, concerns about a hockey stick shortage. Available at: https://toronto.citynews.ca/2020/02/15/amid-coronavirus-outbreak-concerns-about-a-hockey-stick-shortage/ (accessed 29 October 2020).

